# Stepping Stones and Creating Futures Plus: A pilot randomised controlled trial of a co-developed intervention with young South Africans

**DOI:** 10.1371/journal.pgph.0004494

**Published:** 2025-04-23

**Authors:** Andrew Gibbs, Laura Washington, Smanga Mkhwanazi, Esnat Chirwa, Sivuyile Khaula, Nwabisa Jama Shai, Samantha Willan, Neha Batura, Rochelle Burgess, Nangipha Mnandi, Nonhlonipho Simelane, Rachel Jewkes, Jenevieve Mannell

**Affiliations:** 1 Department of Psychology, University of Exeter, Exeter, United Kingdom; 2 Gender and Health Research Unit, South African Medical Research Council, Pretoria, South Africa; 3 Institute for Global Health, University College London, London, United Kingdom; 4 Project Empower, Durban, South Africa; PLOS: Public Library of Science, UNITED STATES OF AMERICA

## Abstract

Marginalised young women and men in South Africa experience and perpetrate high rates of intimate partner violence (IPV), shaped by their contexts and life histories. To address this, we co-developed Stepping Stones and Creating Futures Plus (SSCF+) with young women and men living in marginalised communities to reduce IPV, strengthen livelihoods and improve mental health. We assessed the intervention’s feasibility, acceptability and potential impact, when delivered to small single-gender friendship groups, through a mixed-methods study. We had 30 groups for men and women, and randomly allocated groups to receive the intervention or not. In-depth interviews were conducted post-intervention with n=23 women and n=22 men. Participants were aged 18–30 living in selected communities. We assessed the acceptability of the intervention (participant attendance, overall views) and evidence of change in IPV perpetration (men) and experience (women), livelihoods and mental health 5–6 months post-baseline, and hypothesised mechanisms of change. N=163 men and N=162 women were recruited and we followed up 96.9% men and 100% women at endline. 81% women and 53% men attended at least 70% of sessions, but 3 clusters (10%) ended early. Participants reported the intervention was relevant and enjoyable for them, although they raised some areas for strengthening. At endline, women reported non-significant reductions in physical IPV (aOR0.64, 0.27, 1.53), sexual IPV (aOR0.59, 0.21, 1.65) and severe IPV (aOR0.73, 0.30, 1.75), and significant improvements in livelihoods and mental health. Men reported significantly less physical IPV perpetration (aOR0.38, 0.17, 0.81) and non-significant reductions in sexual IPV (aOR0.77, 0.39, 1.51) and severe IPV (aOR0.86, 0.47, 1.56), as well as improved livelihoods, but no change in mental health. The co-developed SSCF+ intervention was acceptable and showed promise in reducing IPV and strengthening livelihoods, as well as addressing overlapping risk factors. Further research is required to determine its effectiveness. The study was pre-registered at clinicaltrials.gov (NCT05783336).

## Introduction

Young women and men in low- and middle-income countries often live in highly marginalised communities, and frequently experience and perpetrate intimate partner violence (IPV). The high rates of IPV are shaped by the overlapping, mutually reinforcing problems of gender inequality, poor mental health, substance misuse, poverty and other experiences of violence, including in childhood [1,2]. These problems are further located within the framework of weak, and often non-existent, economic and social support systems, alongside the collapse of state systems and histories of colonialism [1].

Shaped by the intergenerational legacies of apartheid and the very high mortality rates of the HIV/AIDS-epidemic [3], young people’s lives in South Africa are characterised by high rates of unemployment, limited education and social exclusion. In South Africa, young people are defined as those aged 15–35 years old, though there is a general focus in research on those aged 15–24 years, as from age 25 years, more start to secure work. At the start of 2023, nationally 33.3% of those aged 15–24 years old, were not in education, employment or training, with this disproportionately impacting those aged 20–24 years, who are black, and who live in urban areas [4]. In recent years, the COVID-19 pandemic led to significant increases in food insecurity and hardship [5]. These have combined to lead to very high rates of HIV, IPV, non-partner violence, alongside poor mental health, high levels of substance misuse and poverty for young people living in marginalised communities [6,7].

Despite the high rates of IPV among young people in marginalised communities, there remains limited evidence of what may be effective interventions to address this. A comprehensive review of interventions to prevent violence highlighted the lack of effective interventions for young people in complex and highly marginalised settings [8]. While a recent systematic review of interventions addressing IPV/dating violence among young people and adolescents, found that while 10 of 11 interventions identified from low- and middle-income countries showed some form of impact on IPV (either for young women’s experience or young men’s perpetration) only two of these 10 were delivered in out-of-school, community settings [9]. The lack of effective IPV prevention interventions among young people out-of-school and not in formal employment, is a major gap given the high rates of violence they experience.

### Stepping Stones and Creating Futures (SSCF)

Since 1998, we have conducted research with a South African adaptation of Stepping Stones to address risk factors for HIV including risky sexual behaviour and gender-based violence [10]. Stepping Stones was originally developed in Uganda in the 1990s to address gender, violence and the HIV-epidemic [11]. We conducted a randomised controlled trial (RCT) with school students (aged 15–26 years old) in rural Eastern Cape schools and showed that the intervention reduced herpes simplex type-2 virus infections among women and men, and men’s perpetration of IPV and problem drinking, but did not significantly reduce women’s experiences of IPV [10].

Recognising the lack of effective IPV prevention interventions for young people out of school and the high levels of unemployment in marginalised communities [12], we focused on these settings. Specifically, we combined Stepping Stones [13] with a livelihoods strengthening intervention, Creating Futures [14].

Between 2016 and 2019 we evaluated Stepping Stones and Creating Futures (SSCF) with young people (ages 18–30 years) living in urban informal settlements in eThekwini Municipality, South Africa, via a RCT [15]. We found two years after receiving the intervention, men were less likely to perpetrate physical IPV, drank less alcohol, and had stronger livelihoods [15]. Women in the intervention – who were not in relationships with the men in the study – reported significantly stronger livelihoods but no other improvements, including no impact on their experiences of IPV [15]. Thus, while the livelihoods component was important for engaging young people in the intervention, it did not show a major advance on the original Stepping Stones intervention for IPV prevention. We identified several areas that needed strengthening. First, while SSCF reduced men’s perpetration of IPV, at endline 41% of men in the intervention reported past year IPV perpetration. Second, there was no impact on women’s experiences of IPV, and for women and men there was no change in measures of mental health or wellbeing. Third, while the SSCF intervention was liked and appreciated by participants, its length ~63 hours, (21 x 3-hour per sessions) impacted delivery cost and led to less-than-ideal attendance – only 22% of men and 31% of women attended 70%+ of sessions [16].

### Co-developing Stepping Stones and Creating Futures Plus

In response to the findings of the SSCF RCT and gaps we identified, alongside our understanding of why interventions may have less than successful outcomes [17], we undertook a co-development process to co-create a new intervention to address IPV. Over three years (2020–2022) we – academics and practitioners - worked with 17 young people aged 18–25 years old, who we refer to as Youth Peer Research Associates (YPRAS), to complete the 6 Steps in Quality Intervention Development (6SQuID) approach [18] in a collaborative method. The young people came from two urban informal settlements in eThekwini Municipality, and a rural community in northern KZN.

To understand the causes of violence in young people’s lives we reviewed previous research and our own research, and supported the YPRAs to reflect on the underlying drivers of their experience and perpetration of IPV, and developed locally relevant models to explain this. Methods included an artefact creation process, similar to photo-elicitation [19], with the team thematically analysing the data produced and sharing back to YPRAs, and creating storylines of young people’s lives [17]. To co-develop a theory of change we used a problem-tree methodology, whereby YPRAs identified the roots (causes) of the trunk (problem – violence) and the branches and leaves (consequences). This was then flipped over to identify solutions. We then spent time working with the YPRAs to identify strategies to bridge the gap between the causes and potential futures they envisaged.

The research and practitioner team then took this to create an intervention manual based on the strategies we had agreed, building off our previous research and experience in South Africa and globally [20]. Sessions were tested with the YPRAs, with them reflecting on the extent they related to the ToC and suggesting changes, before the manual was ‘pre-tested’ with a small group of young people before finalisation. The final co-created intervention was called Stepping Stones and Creating Futures Plus (SSCF+) recognising the foundational work it built on.

The co-developed SSCF+ intervention comprises 15 sessions, with each session approximately 3-hours long (total ~45 hours), delivered primarily to single-gender friendship groups (see S1 Table for detail). The intervention methodology is driven by Freire’s [21] adult-education approach, emphasising drawing out young people’s lived realities, and through discussion and reflection, generating critical thinking about topics of importance for young people. Critical thinking is developed through facilitated group discussions focused on of issues of importance, providing space for young people to see connections between their individual stories and collective experiences and identify broader causes [22]. This is backed up by a mixture of skills building and practice of these skills in groups. The curriculum focuses on intervening at the nexus of poor mental health, poverty, hunger and IPV through supporting young people to develop their livelihoods, improve their ability to relate positively with others, address gender inequality and increase feelings of wellbeing.

Several key threads run across intervention sessions. First, there is an emphasis on working with, and strengthening, friendship groups. This builds on young people’s views that friendship groups, while critical for survival in challenging contexts, were also sources of tension and conflict. To achieve this friendship groups are recruited through a revised respondent-driven sampling methodology – essentially asking a suitable person to recruit their friends who met eligibility criteria. These small friendship groups form the groups the intervention is targeted to. In sessions, practical skills, such as active listening are taught and practiced in a manner directed at strengthening friendship group dynamics. Activities included sharing life stories (often for the first time) in the group and promoting active, non-judgemental listening in relation to these, and building group cohesion.

Second, supporting improved wellbeing runs throughout sessions. Participants are taught and practice basic short techniques to reduce stress – drawn from Self-Help Plus and Problem Management Plus [23,24]. Strategies taught and practiced include focused breathing and closing their eyes to calm themselves. Additionally, aspects of narrative therapy are built in throughout sessions, particularly when participants share life stories, often of adversity and disappointment, which are reflected back to participants in terms of resilience [25].

Third, understanding and addressing gender inequalities is central to the intervention. Activities are designed to encourage people to reflect on how gender norms impact young people’s daily lives, relationships (intimate, familial and friendships) and how violence in relationships occurs and promote change to these through a mixture of skills building and reflection. To strengthen the intervention, we included two mixed-gender peer group sessions, bringing together male and female groups, these are designed to provide space for young people to discuss gendered issues, including abuse, in a supported space and for men to build empathy towards women.

Sessions are designed to be delivered by same gender trained peer facilitators – with mixed gender-sessions led by a fe/male pair of facilitators - ideally coming from the communities where the intervention is being delivered and thus also delivered in languages used by participants (in this case primarily isiZulu, with some English words/phrases). Facilitators require five weeks of training. Given the small group sizes, intervention venues range from people’s shacks, to small community spaces. Sessions are designed to be held twice a week, at a time suiting each group, and provide flexibility for sessions to be re-arranged if needed. In sessions, participants receive basic food and drink. Additionally, participants receive a R100(US$5) travel allowance for each session attended (maximum possible: R1500[US$75]).

We conducted a mixed-methods study to understand: 1) is the new intervention acceptable to young people, and 2) does SSCF+ show evidence of promise on key hypothesised mechanisms, such that it would be suitable for evaluation in a larger efficacy trial.

## Materials and methods

### Ethics statement

This study received ethical approval from the South African Medical Research Council’s Human Research Ethics Committee (EC023–10/2022) and University of Exeter’s Department of Psychology Ethics Committee (570602), and UCL provided reciprocal approval based on the University of Exeter’s approval, prior to study commencement. Participants provided written informed consent prior to their involvement. Additional informed consent was provided by those recruited into the qualitative study. The study was pre-registered at clinicaltrials.gov (NCT05783336), and we follow the CONSORT guidance on reporting of pilot and feasibility trials (S1 Consort).

**Fig 1:**
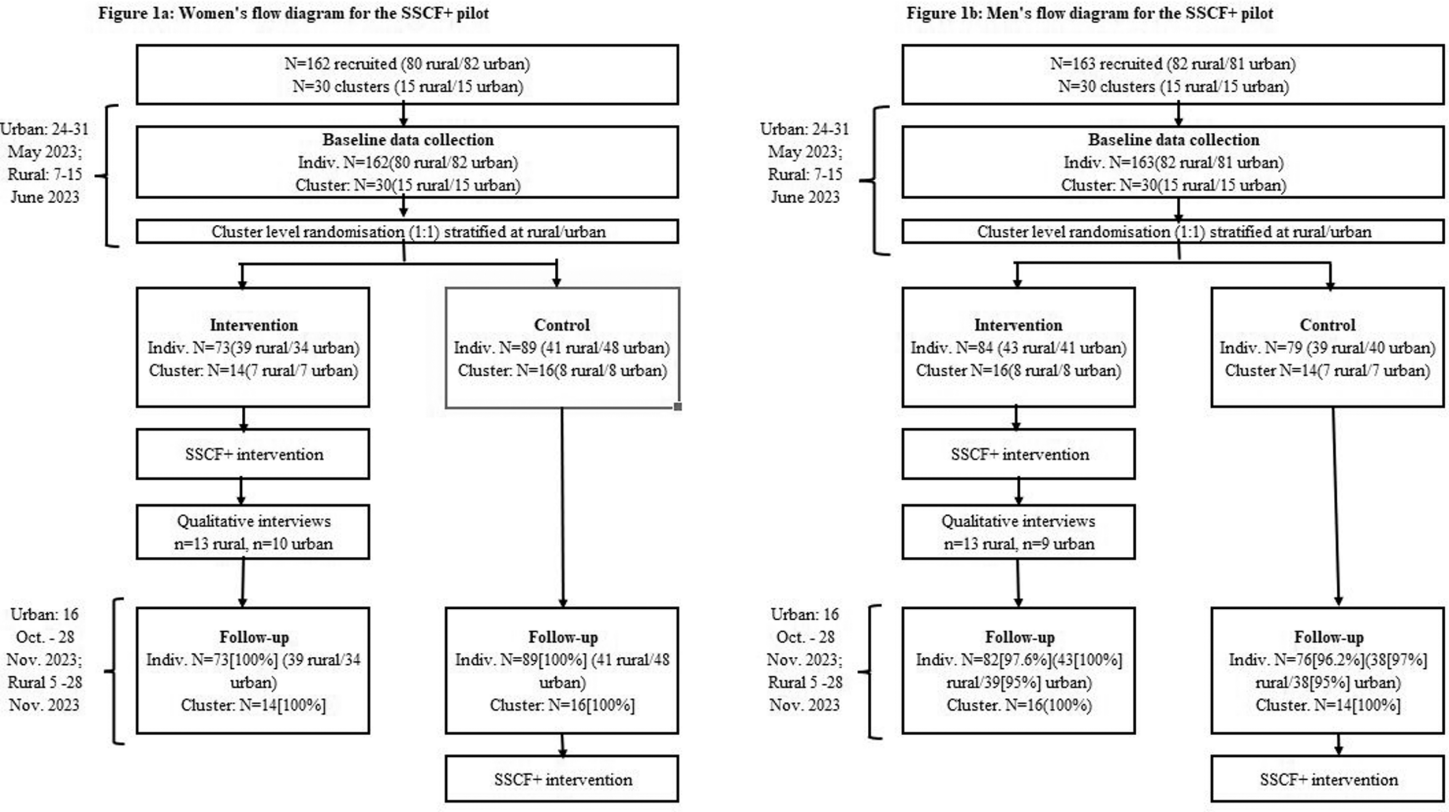
CONSORT flow diagram for women and men in the study.

We conducted a mixed-methods pilot-randomised acceptability and feasability study, with the post-test assessment 5-months after baseline. To assess intervention promise, we had an equal number of comparison clusters and randomised study participant friendship groups, per gender and community, to receive the intervention immediately, or delayed. The delayed intervention groups were offered SSCF+ after the last assessment. We used the Excel random number generator to randomly allocate clusters to each arm after the baseline interview. Qualitative interviews were conducted in isiZulu with participants in the intervention arm, shortly after the intervention had been completed (Fig 1).

Participants came from two sites. In the eThekwini Municipality, in KwaZulu-Natal Province (KZN), we identified two urban informal settlements where we could safely work. Both had limited access to formal water and electricity supply, as well as high levels of food insecurity. We also worked in one community in northern KZN province, a 6–7 hour drive from eThekwini Municipality. In this community, there was little agriculture or other work, and most people looked to migrate to urban communities, including eThekwini Municipality, for employment.

Participant eligibility was being aged 18–30 years at recruitment. We initially wished to focus on those aged 18–25 years, given the high rates of IPV, unemployment and challenges they face, but friendship groups extended beyond this neat approach, and we increased the age limit to 30 years. Additional inclusion criteria were, normally living in the community where we were recruiting, able and willing to provide written informed consent and able to communicate in one of the study languages (English, isiZulu, isiXhosa). IPV perpetration/experience was not an inclusion criterion for two reasons: first, given we recruited in friendship groups, it would have been ethically problematic to include/exclude on this. Second, the factors shaping violence perpetration/experience often cluster in friendship groups, and require friends as a group to change to impact on these.

We recruited participants in small groups via a chain-recruitment method. The team’s facilitators – many of whom had been involved in co-developing SSCF+ and came from the communities we operated in - identified young people meeting the eligibility criteria, and asked them to recruit up to 10 friends who met the same eligibility criteria. These friendship groups formed clusters, to which the intervention could be delivered.

Friendship groups were invited to attend a formal study introduction, with a separate validation of eligibility undertaken by the research team’s fieldworkers. Written informed consent was sought from participants. Baseline recruitment began on Wednesday 24 May 2023 and was completed on 15 June 2023.

### Outcomes

Acceptability of SSCF+ for participants was assessed in two ways. First, we assessed young people’s attendance at the intervention through attendance registers. We estimated the proportion of participants who provided informed consent, who attended at least one session and the proportion of participants who attended 11 or more sessions (73%+) sessions. Second, we explored young people’s views on the major changes and adaptations of SSCF+, specifically its relevance to their lives, intervening with small friendship groups, the mental health components and the mixed-gender sessions. This was done via in-depth interviews with same-sex, trained researchers. Interviews were conducted in locations where audio privacy could be assured and focused on young people’s experiences of the intervention and what they would like changing, and whether there were aspects they struggled with. Participants self-selected into the qualitative interviews. Interviews were conducted in isiZulu, recorded, translated and transcribed into English, ensuring participants’ identities were anonymised (e.g., through pseudonyms, changing community names and other identifying features) and then subjected to thematic analysis [26] focused on overall experience and the themes of friendship groups, mental health, and mixed-gender sessions.

To assess potential intervention effectiveness and whether SSCF+ was suitable for a future efficacy trial, we assessed a range of quantitative measures linked to hypothesised pathways of change and outcomes. We pre-specified three primary outcomes - past 6-month physical IPV perpetration (men) and experience (women) assessing five items related to physical violence (e.g., slap, hit); past 6-month sexual IPV perpetration (men) and experience (women) using three items (e.g., coerced sex, too drunk to consent). Both these were considered as binary, with any positive response to an item, leading people to be classified as having experienced/perpetrated IPV; and past 6-month severe physical and/or sexual IPV perpetration (men) and experience (women). Severe IPV was understood as two or more experiences of physical and/or sexual IPV (see Table 1 for details). These were assessed using a South African adaptation of the WHO Violence Against Women Scale [27], and for men the revised approach within the UN Multi-Country Survey (UNMCS). We pre-specified a range of secondary outcomes covering other forms of IPV experience/perpetration, mental health, substance misuse, and livelihoods – all hypothesised pathways to change, drawing on validated scales, which had been previously used in South Africa (see Table 1 for details). All primary and secondary outcomes were registered with clinicaltrails.gov). We also report a series of tertiary outcomes in Table 1.

**Table 1:** Outcomes used to assess potential impact of the intervention.

	Source	Indicator	Number of items	Cronbach Alpha - Women	Cronbach Alpha - Men	Method of scaling	Hypothesised direction of change
**Primary Outcomes**							
Physical IPV in past 6m	WHO VAW Scale [27], adapted and widely used in South Africa	One or more episode of physical IPV in the past 6 months (women experience, men perpetrate)	5			Binary	Decrease
Sexual IPV in past 6m		One or more episode of sexual IPV in the past 6 months (women experience, men perpetrate)	3			Binary	Decrease
Severe IPV in past 6m		Two or more episodes of physical and/or sexual IPV in the past 6 months (women experience, men perpetrate)	8			Binary	Decrease
**Secondary** + **Tertiary Outcomes**							
***IPV, gender norms - tertiary outcomes, not pre-specified***							
Emotional IPV in past 6m	WHO VAW Scale [27], adapted and widely used in South Africa	One or more episode of emotional IPV in the past 6 months (women experience, men perpetrate)	5			Binary	Decrease
Economic IPV in past 6m		One or more episode of economic IPV in the past 6 months (women experience, men perpetrate)	5			Binary	Decrease
Gender inequitable attitudes>= less equitable gender attitudes	Adapted from Gender Equitable Men’s Scale [28]	Agreement with statements on gender attitudes (4-point Likert scale, more inequitable)	12	0.82	0.76	Score	Decrease
Controlling behaviour>= more controlling	Adapted from SRP Scale [29] for use in South Africa	Experiences of control in primary relationships by male partner (men perpetrate and women experience)	13	0.88	0.82	Score	Decrease
Communication>= better	Communications Pattern Questionnaire	Communication skills in main intimate relationships, continuous	11	0.67	0.74	Score	Increase
**Livelihoods**							
***Livelihoods - Secondary pre-specified***							
Any earnings in past 4 weeks	Used in previous SSCF trial	Earnings in past month (ZAR)	1			Binary (0 versus any)	Increase
Amount of earnings in past 4 weeks	Used in previous SSCF trial	Earnings in past month (ZAR)	1			Score	Increase
Any savings in past 4 weeks	Used in previous SSCF trial	Savings in past month (ZAR)	1			Binary (0 versus any)	Increase
Amount of savings in past 4 weeks	Used in previous SSCF trial	Savings in past month (ZAR)	1			Score	Increase
Food insecurity score high=more insecure	Coates et al [30] - used globally	Household food insecurity in the past 4 weeks	3			Score	Decrease
***Livelihoods - Tertiary, not pre-specified***							
Worked in past 3 m	Used in previous SSCF trial	Worked in the past three months	1			Binary (yes versus no)	Increase
Livelihood activities score high=more active	UNMCS Men and Violence Study	Livelihood activities	7	0.80	0.71	Score	Increase
How easy to find R200 in emergency	Used in previous SSCF trial	Ability to access R200 in an emergency (very difficult or somewhat difficult, compared with somewhat easy, easy)	1			Binary (hard, versus easy, very easy)	Increase
Borrow food or money	Used in previous SSCF trial	Need to borrow food or money in the past 4 weeks as not having enough (once or twice, versus more often)	1			Binary (never, once or twice, versus three or more times)	Decrease
Taken something that was not yours	Used in previous SSCF trial	Stealing in the past 4 weeks because of hunger (never, once versus two or three, or more often)	1			Binary	Decrease
**Mental Health and Substance Use**							
***Mental health - secondary outcomes, pre-specified***							
Depressive symptoms>= more	CESD20 Scale [31]	Past 2 weeks depressive symptoms, continuous	20	0.87	0.81	Score	Decrease
Post traumatic symptoms>=more	HTQ [32]	Past week symptoms of post-traumatic distress, continuous	15	0.93	0.90	Score	Decrease
Experience of stress due to lack of work high=more stress	IMAGES Study [33]	Stress related to lack of work and unemployment, continuous	4	0.80	0.76	Score	Decrease
Experience of shame due to lack of work high=more shame	IMAGES Study [33]	Shame related to lack of work and unemployment, continuous	4	0.65	0.49	Score	Decrease
**Mental health - tertiary outcomes, not pre-specified**							
Anxiety score high=more anxiety	GAD7 [34]	Past two weeks symptoms of anxiety, continuous	7	0.79	0.82	Score	Decrease
Problem drinking	AUDIT-C [35]	Past 6 month binge drinking using recommended cuts	3			Binary (4+)	Decrease
Alcohol use (score)	AUDIT-C [35]	Past 6 month alcohol consumption (score>=more)	3			Score	Decrease

Notes: AUDIT-C - Alcohol use disorders identification test for consumption; GAD7 - Generalised Anxiety Disorder Scale; CESD20 - The Center for Epidemiological Studies-Depression; HTQ20 - Harvard Trauma Questionnaire; SSCF - Stepping Stones and Creating Futures; IMAGES Study - International Men and Gender Equality Survey

Questionnaires were self-completed on a cellphone, using KoboBox with audio support, in either English, isiZulu or isiXhosa. The system had inbuilt logic and range checks. A fieldworker was nearby if participants required support. At endline, we tracked participants using contact information and friendship networks. If we found them in person, they self-completed the questionnaire for a second time, whereas if we could only contact them over the phone, we conducted a shorter questionnaire telephonically with a fieldworker reading out questions.

Given this was a feasibility study, we conducted a power calculation for our known sample size (N=160 male, N=160 female), based on a range of assumptions. We did this power calculation based on data from a previous study where past 6 month IPV prevalence was approximately 30% [36]. We provided a range of estimates for the power of our sample, showing differences between numbers of clusters, people/cluster, and intraclass correlation coefficients (ICCs), with power ranging from 0.45 to 0.61 (S2 Table).

### Statistical methods

We recognise that this is a small acceptability study and not a randomised controlled trial (RCT), however, we analysed the data and report the findings following the approaches of an RCT in order to maximise our clarity about what the findings showed, and reduce the impact of bias as far as possible. The primary analysis was conducted at an individual level and followed an intention to treat (ITT) approach. We first assessed missingness of scales at baseline and endline. Missing item responses range from 2%-11% among men and 2%-8% among women, although almost all participants responded to more than 90% of items for each scale. We used mean item imputation at the individual level to retain sample size, but excluded those who responded to no items within a scale. Those lost to follow up at endline were excluded in the analysis.

Frequencies and percentages summarized binary or categorical measures by intervention group. Mean and standard deviation or median and interquartile ranges (IQR) were used for continuous measures. Chi-square tests and independent sample t-tests were used to perform bivariate analysis. We used mixed effects models to assess intervention effect on continuous outcomes, taking into account variations between clusters. For binary outcomes, generalized estimating equations (GEE) were used with an exchangeable correlation matrix and robust standard errors. All models were adjusted for the baseline measure of the outcome.

Supplementary analyses were conducted. First, adjusting for participant location (urban/rural). Second, a per protocol analysis compared those who attended at least 73% (11+) of sessions, compared to control. All analysis was done in Stata 17 and significance testing was at 5%.

## Results

Fig 1 shows the participant flow for women and men. Participants were recruited 24–31 May 2023 in urban informal settlements and 7 to 15 June 2023 in rural communities. In total N=162 women were recruited in 30 clusters, with the most common cluster size being 5 (range 4–8). For men, N=163 were recruited in 30 clusters. The most common cluster size was 5 (range 4–7).

The intervention delivery was successful. We recruited friendship groups and established a flexible timetable to accommodate the needs of individuals in each group. We specified each group should meet twice weekly, but at a time and a place that suited them and their facilitator. We wanted all groups to cover the same sessions each week, as this made it easier for us to support the mostly inexperienced facilitators and ensure better quality delivery. Most women’s groups chose to meet between 10 am and 1 pm on the same day each week, while the men’s groups had a more variable timetable to accommodate once-off job opportunities. Facilitators met on Fridays with the Project Empower leads, to review session delivery, troubleshoot problems and plan for the next week’s sessions. The intervention was in the main delivered well, but despite training and support offered, some facilitators struggled to balance their role as group leaders/facilitators with their ongoing relationships with peers.

### Acceptability

Overall participant attendance was high. Among women, 90%(n=66) of those who were enrolled into the study and randomised to the intervention attended at least one session and 81%(n=59) attended 11 or more sessions (mean number attended 11.8 sessions). One cluster was terminated early, with only two (of five) women attending one session. Among men, 86% (n=70) of those offered the intervention attended at least one session, and 53% (n=43) attended 11 or more sessions (mean number attended 8.9 sessions). Little or no intervention was delivered in two male clusters in the urban communities, but these men were followed up in data collection. One group felt the intervention was not what they wanted, while another group attended one session, but because of alcohol use by all of the men in this group, struggled to attend another, despite a range of options around times and days being offered to them.

Qualitative data were collected from 23 women (n=13 rural, n=10 urban) and 22 men (n=13 rural, n=9 urban) after the intervention had been completed. Basic demographic information is provided in Table 2, and many had not completed high school education. Almost all participants described SSCF+ as being focused on the challenges young people faced: “*All the sessions were relevant because they are all a part of life.” (Sindiswa, female, rural)*. They described learning a mixture of skills, from how to write CVs, to how to communicate with others. A few reported, however, that some sessions were not relevant to them, often because they had not experienced the issue – for instance debt. Others suggested the need to include new sessions, such as on how to use new technology.

**Table 2:** Socio-demographics of participants involved in qualitative data collection.

Name^a^	Male/Female	Urban/Rural	Age	Education
Sandile	M	R	24	Completed high school
Themba	M	R	24	Grade 10
Melukhule	M	R	21	Completed high school
Lungelo	M	R	26	Completed high school
Spha	M	R	27	Didn’t complete high school
Mkhabo	M	R	23	Grade 12 - not completed
Siyamanthi	M	R	25	Completed high school
Wandile	M	R	23	Grade 10
Hazard	M	R	20	Completed high school
Simphiwe	M	R	23	Grade 10
Sandile	M	R	25	Completed high school
King	M	R	21	Completed high school
KimKing	M	R	23	Completed high school
Slindile	F	R	20	Completed high school
Simphiwe	F	R	27	Didn’t completed high school
Bayanda	F	R		Completed high school
Lubanzi	F	R	28	Completed high school
Sphereshi	F	R	23	Not clear
Nonhle	F	R	25	Grade 11
Thando	F	R	26	Completed high school
Pretty	F	R	19	Completed high school
Sindiswa	F	R	24	Completed high school
Emihle	F	R	25	Grade 11
Mantombana	F	R	25	Didn’t complete high school
Zanele	F	R	27	Completed high school
Wendy	F	R	22	Grade 11
Hope	F	R	25	Completed high school
Lungelo	M	U	19	Completed high school
Dash	M	U	26	Completed high school
Jesu	M	U	24	Started university but dropped out
Boy	M	U	24	Grade 11
Mjebula	M	U	26	Grade 11
Nhlanzeko	M	U	24	Completed high school
Sthe	M	U	27	Grade 11
Qiniso	M	U	18	Completed high school
Sphamandla	M	U	18	Grade 10
Luyanda	M	U	20	Completed high school
Athandile	F	U	19	Completed high school
Teddy	F	U	21	Grade 11
Nelisiwa	F	U	18	Completed high school
Stharara	F	U	26	Grade 11
Mancane	F	U	30	Grade 11
Lusanda	F	U	18	Completed high school
Boh	F	U	23	Grade 12 - not completed
Anita	F	U	18	Grade 10
Mbhalenhle	F	U	19	Completed high school
Lihle	F	U	24	Didn’t complete high school
Nowe	F	U	20	Grade 8
Nobuhle	F	U		Grade 10

^a^All names are pseudonyms

Many described liking the participatory nature of SSCF+, with participants enjoying the opportunities for role plays (Mancane, female, urban), and discussions. One participant contrasted the learning approach in school, and what they were taught at home, with SSCF+ where the emphasis was on discussion and reflection:

*Things that we were not taught at home and at school ehh you know parents have that thing sometimes, where I do not know if they are scared of you, or they just do not want you to know certain things…(Dash, male, urban)*

Facilitators played an important role in creating a space where everyone was equal and could speak openly:

*I think the way the facilitators taught us, they gave themselves time, they made sure that everyone participates. Everyone enjoyed being in class, they treated us equally. They told us that in class no one was better than the other, and there are no wrong or right answers.* (Sandile, male, rural)

Many of the participants talked positively about the intervention working with friendship groups: “*I felt very comfortable.” (Nowe, female, urban)*. Some described being able to open up more quickly in friendship groups than if they had been in larger groups: “*It’s much better [in a friendship group] because I am a shy person and if it’s new people, or people I don’t know, it was going to be difficult for me because I am a shy person…”* Mbhalenhle (female, urban). Participants also described how the intervention helped improve their friendships:

*It was a good thing because I have learnt a lot from my friends here. We might spend most of our time together, but I did not know that in an intimate way. So, it gave me the chance to know them better. (Lungelo, male, rural).*

The main challenge with recruiting and delivering to small friendship groups was that we had imposed an age restriction (18–30 years). This broke-up some friendship groups: “*They [other friends] wished to participate but the problem was that some of them did not qualify” (Stharara, female, urban).*

A new component introduced to SSCF+ were activities focused on developing coping skills, drawn primarily from Self-Help Plus and Problem Management Plus [23,37]. Overall, women and men tended to enjoy these and felt they helped them: “*The breathing exercise helped me to get my mind off a lot of things and made me be able to focus and think more cl*early.” (King, male, urban). Some described using these skills outside of sessions:

“*Yes, yeah, I sometimes did it. When I found myself angry and I do want to talk or pick a fight with another person. It helps me in that way. Or when I feel my heart beating fast.” (Boy, male, urban).*

While most described liking and using these activities, some only did them in sessions and not outside. While a few described how closing their eyes led to traumatic flash-backs:

“*I* *did not feel comfortable with that exercise, because every time when I closed my eyes my boyfriend who used to abuse me would reappear. When I closed my eyes, I would see his picture and all the terrible things he did to me. (Stharara, female, urban)*

Most of the interviewees spoke positively about the two mixed-peer group sessions: “*It was fun, I enjoyed them a lot*.” (Silindle, female, rural). They described learning a lot from listening to the other gender, particularly around sex and relationships:

“*Yes, when we did the joys and pleasures of sex, we all really enjoyed that session. It was very insightful because we got to learn about what women don’t like” (Hazard, male, rural)*.

For some participants, the mixed group sessions encouraged them to take other views:

“*We were discussing how women feel in their relationships, you know things like that. I got to understand their perspectives, you know things like that.” (Jesu, male, urban)*.

However, some women found mixed groups challenging. A couple of female participants described not feeling happy being in groups with men: “*I was not completely comfortable with the role play with boys, but I felt compelled to do it.” (Sphereshi, female, rural)*. Another described being shy at first, but then liking the session:

“*But the first time I feared them [the men]. There were some guys that I was afraid of, but the following day was alright, and everything was good.” (Stharara, female, urban)*.

A few women, however, felt the men had insulted them. In one session, where women and men describe what they like about bodies, some women felt judged:

“*The one about boys judging us and telling us about the things they don’t like about us...Them telling us that we have dimples on our bums and that we have big breasts and they don’t like that.”* (Bayanda, female, rural).

Overall, SSCF+ was seen as something that was relevant to young people’s lives, engaging and the intervention approach with small groups of friends was well liked and enabled friendships to be strengthened.

### Quantitative outcomes

At baseline (Table 3) women’s mean age was 23.3 years and almost half (43.2%) had passed high school (matric). Two-thirds (66.7%) reported they had a partner, who they did not live with, almost a fifth (17.0%) reported no current relationship, and 16.3% reported they were married or lived with a partner. About one third (34.0%) lived in a shack, 30.8% in a single room, and a quarter (23.8%) in a formal house. There were no significant differences between socio-demographic characteristics and arm allocation, though there was a suggestion that women in the intervention were somewhat younger than those in the control arm (less than 20: 23.3% v 11.2%, p=0.098).

**Table 3:** Socio-demographic and outcome measures at baseline for women and men, compared by arm allocation.

			Women					Men			
		**Overall**	**Control**		**Intervention**	**p-value**	**Overall**	**Control**		**Intervention**	**p-value**
**Socio-demographics**		**N=162**	**N=89**		**N=73**		**N=163**	**N=79**		**N=84**	
Age											
18-19yrs		27 (16.7%)	10 (11.2%)	17 (23.3%)		0.098	12 (7.4%)		7 (8.9%)	5 (6.0%)	0.11
20-24yrs		77 (47.5%)	47 (52.8%)	30 (41.1%)			86 (52.8%)		47 (59.5%)	39 (46.4%)	
25-30yrs		58 (35.8%)	32 (36.0%)	26 (35.6%)			65 (39.9%)		25 (31.6%)	40 (47.6%)	
Education											
Grade 10 or below		34 (21.0%)	19 (21.3%)	15 (20.5%)		0.99	44 (27.0%)		22 (27.8%)	22 (26.2%)	0.58
Grade11		39 (24.1%)	22 (24.7%)	17 (23.3%)			26 (16.0%)		14 (17.7%)	12 (14.3%)	
Grade 12		19 (11.7%)	10 (11.2%)	9 (12.3%)			13 (8.0%)		8 (10.1%)	5 (6.0%)	
Passed matric		70 (43.2%)	38 (42.7%)	32 (43.8%)			80 (49.1%)		35 (44.3%)	45 (53.6%)	
Relationship											
Partner, not living together		103 (67.3%)	55 (65.5%)	48 (69.6%)		0.72	123 (76.9%)		66 (83.5%)	57 (70.4%)	0.14
Partner, living together		24 (15.7%)	15 (17.9%)	9 (13.0%)			16 (10.0%)		6 (7.6%)	10 (12.3%)	
No relationship		26 (17.0%)	14 (16.7%)	12 (17.4%)			21 (13.1%)		7 (8.9%)	14 (17.3%)	
Housing											
Bond/RDP house		36 (23.1%)	20 (23.3%)	16 (22.9%)		0.49	30 (18.5%)		11 (13.9%)	19 (22.9%)	0.38
Shack/informal dwelling		53 (34.0%)	25 (29.1%)	28 (40.0%)			45 (27.8%)		23 (29.1%)	22 (26.5%)	
Single Room		48 (30.8%)	29 (33.7%)	19 (27.1%)			78 (48.2%)		39 (49.4%)	39 (47.0%)	
Other		19 (12.2%)	12 (14.0%)	7 (10.0%)			9 (5.6%)		6 (7.6%)	3 (3.6%)	
**Primary Outcomes**											
Physical IPV in past 6m	Yes	65 (40.6%)	36 (41.4%)	29 (39.7%)		0.83	54 (33.1%)		22 (27.8%)	32 (38.1%)	0.16
Sexual IPV in past 6m	Yes	36 (22.6%)	18 (20.7%)	18 (25.0%)		0.52	31 (19.4%)		13 (16.7%)	18 (21.4%)	0.44
Severe IPV in past 6m	Yes	56 (35.0%)	31 (35.6%)	25 (34.2%)		0.85	43 (26.4%)		22 (27.8%)	21 (25.0%)	0.68
***IPV, gender norms - tertiary outcomes, not pre-specified***											
Emotional IPV in past 6m	Yes	91 (57.2%)	46 (52.9%)	45 (62.5%)		0.22	93 (57.1%)		40 (50.6%)	53 (63.1%)	0.11
Economic IPV in past 6m	Yes	69 (43.4%)	40 (46.0%)	29 (40.3%)		0.47	79 (48.5%)		31 (39.2%)	48 (57.1%)	0.022
Gender inequitable attitudes>= less equitable gender attitudes		24.6 (5.7)	25.5 (5.7)	23.6 (5.5)		0.04	27.4 (5.3)		28.0 (4.9)	26.8 (5.6)	0.15
Controlling behaviour>= more controlling		28.2 (6.9)	28.6 (6.7)	27.7 (7.2)		0.44	29.5 (6.1)		29.5 (5.8)	29.5 (6.3)	0.95
Communication>= better		20.4 (5.9)	21.0(5.8)	19.5(6.0)		0.11	20.7 (6.2)		20.3(6.1)	21.1(6.4)	0.427
**Livelihoods**											
***Livelihoods - Secondary pre-specified***											
Any earnings in past 4 weeks	Yes	113 (72.9%)	67 (77.0%)	46 (67.6%)		0.19	140 (88.1%)		67 (87.0%)	73 (89.0%)	0.7
Amount of earnings in past 4 weeks			300(1-400)	300 (0-500)		0.78			300 (100-500)	350 (150-500)	0.81
Any savings in past 4 weeks	Yes	80 (51.0%)	41 (47.1%)	39 (55.7%)		0.28	90 (57.3%)		45 (61.6%)	45 (53.6%)	0.31
Amount of savings in past 4 weeks			0 (0-150)	20 (0-150)		0.37			80 (0-200)	15 (0-200)	0.28
***Livelihoods - Tertiary, not pre-specified***											
Worked in past 3 m	Yes	22 (13.9%)	14 (16.3%)	8 (11.1%)		0.35	51 (31.9%)		26 (32.9%)	25 (30.9%)	0.78
Livelihood activities score high=more active		11.3 (6.3)	12.3 (4.7)	10.0 (6.3)		0.011	11.8 (4.8)		11.7 (5.3)	11.8 (4.3)	0.85
How easy to find R200 in emergency	Very easy	6 (3.9%)	2 (2.4%)	4 (5.7%)		0.28	5 (3.1%)		4 (5.1%)	1 (1.2%)	0.15
Borrow food or money	>=2	43 (27.4%)	24 (27.9%)	19 (26.8%)		0.87	40 (24.8%)		20 (25.3%)	20 (24.4%)	0.89
Taken something that was not yours	Yes	59 (37.3%)	33 (38.4%)	26 (36.1%)		0.77	63 (39.9%)		30 (38.0%)	33 (41.8%)	0.63
**Mental Health and Substance Use**											
***Mental health - secondary outcomes, pre-specified***											
Depression score high= more depressed		21.0 (11.2)	22.3 (11.4)	19.6 (11.0)		0.13	17.5 (9.0)		16.7 (9.3)	18.3 (8.6)	0.26
PTS symptoms experience high= more		15.4 (9.5)	16.5 (11.2)	14.1 (9.5)		0.15	12.3 (8.7)		12.3 (9.4)	12.4 (8.0)	0.91
Experience of stress due to lack of work high=more stress		12.6 (2.8)	12.8 (2.6)	12.5 (2.9)		0.49	12.1 (2.7)		12.1 (2.4)	12.1 (2.9)	0.96
Experience shame due to lack of work high=more shame		10.4 (2.5)	10.6 (2.3)	10.2 (2.7)		0.27	10.4 (2.3)		10.2 (2.0)	10.5 (2.6)	0.38
**Mental health - tertiary outcomes, not pre-specified**											
GAD score high=more anxiety		6.8 (4.0)	7.5 (4.8)	5.9 (4.0)		0.027	6.5 (4.8)		6.2 (5.2)	6.7 (4.5)	0.53
Problem drinking	Yes	43 (27.2%)	19 (22.1%)	24 (33.3%)		0.11	48 (29.6%)		24 (30.4%)	24 (28.9%)	0.84
Audit score			0.0 (0.0–2.0)		0.5 (0.0–4.0)	0.14		2.0 (0.0–4.0)		2.0 (0.0–4.0)	0.3

Comparing women’s outcomes at baseline by arm allocation (Table 3), those in the control arm held more inequitable gender attitudes and symptoms of anxiety than those in the intervention arm (p=0.04), while also reporting more livelihood activities (p=0.01).

Among men, at baseline (Table 3) mean age was 23.8 years, and half (49.1%) had completed high school. Three-quarters (76.9%) reported having a non-cohabiting partner, while only 10% reported cohabiting. Half (48.2%) lived in a single room, a quarter (27.8%) in a shack, and a fifth (18.5%) in a formal house. There were no differences by arm for men’s socio-demographic characteristics. Randomisation generally resulted in balanced arms for men (Table 3). However, a higher proportion of men in the intervention arm reported past 6-month perpetration of economic IPV compared to those in the control arm (p=0.022).

Follow-up rates (Fig 1) at endline were high. Among women (Fig 1a), follow-up was 100%(N=162). While most (94.4%) women completed the full questionnaire, more women in the control arm completed the short questionnaire (I:1.4% v C:9.0%, p=0.042, Fishers Exact). Follow-up among men was high (96.9%, N=158, Fig 1b), and most completed the full questionnaire (91.8%), with no difference by arm.

#### Women - outcomes.

Among women at endline those randomised to receive SSCF+ reported no significant differences in their past 6-month experience of IPV (Table 4). However, adjusted models all indicated non-significant reductions for IPV experience for women in SSCF+. Specifically, past 6-month physical IPV experience (aOR0.64, 95%CI 0.27, 1.53), sexual IPV (aOR0.59, 95%CI 0.21, 1.65) and severe IPV (aOR0.73, 95%CI 0.30, 1.75) were all lower. Other measured outcomes related to IPV and gender practices showed improvements among those in the intervention arm in the direction expected, with a significant reduction in women’s experiences of controlling behaviours reported (β-1.72, 95%CI -3.33, -0.11).

**Table 4:** Endline outcome measures for women.

	Women				
	**Control**	**Intervention**	**p-value**	**Model 1**	
	**N=89**	**N=73**		**aOR/B (95%CI)**	**p-value**
**Primary Outcomes**					
Physical IPV in past 6m	41 (46.1%)	26 (36.1%)	0.200	0.64 (0.27, 1.53)	0.314
Sexual IPV in past 6m	27 (30.3%)	17 (23.6%)	0.340	0.59 (0.21, 1.65)	0.317
Severe IPV in past 6m	37 (41.6%)	25 (34.7%)	0.370	0.73 (0.30, 1.75)	0.480
***IPV, gender norms - tertiary outcomes, not pre-specified***					
Emotional IPV in past 6m	51 (63.0%)	41 (57.7%)	0.510	0.72 (0.26, 1.97)	0.525
Economic IPV in past 6m	48 (59.3%)	34 (48.6%)	0.190	0.70 (0.32, 1.50)	0.357
Gender inequitable attitudes>= less equitable gender attitudes	24.9 (5.1)	23.2 (5.3)	0.039	−0.63 (−1.92, 0.67)	0.343
Controlling behaviour>= more controlling	30.3 (7.7)	27.9 (7.4)	0.051	−1.72 (−3.33, −0.11)	0.037
Communication>= better	21.5(6.5)	20.2(6.1)	0.200	`−0.87 (−2.75, 1.02)	0.366
**Livelihoods**					
***Livelihoods - Secondary pre-specified***					
Any earnings in past 4 weeks	61 (71.8%)	53 (82.8%)	0.120	2.11 (1.04, 4.28)	0.038
Amount of earnings in past 4 weeks	200 (0–500)	325 (150–900)	0.014	0.98 (0.05, 1.90)	0.039
Any savings in past 4 weeks	36 (45.6%)	47 (70.1%)	0.003	3.29 (1.14, 9.46)	0.027
Amount of savings in past 4 weeks	0 (0–200)	150 (0–300)	0.003	1.17 (0.34, 1.99)	0.006
Food insecurity score high=more insecure	7.4 (2.5)	6.4 (2.8)	0.014	−0.92 (−1.64, −0.21)	0.011
***Livelihoods - Tertiary, not pre-specified***					
Worked in past 3 m	20 (22.5%)	31 (43.7%)	0.004	2.86 (1.34, 6.11)	0.007
Livelihood activities score high=more active	12.2 (5.5)	12.0 (5.1)	0.820	1.13 (−0.37, 2.62)	0.140
How easy to find R200 in emergency	0 (0.0%)	2 (2.8%)	0.110	n/a	
Borrow food or money	37 (41.6%)	15 (21.1%)	0.006	0.34 (0.15, 0.76)	0.009
Taken something that was not yours	46 (51.7%)	25 (35.2%)	0.037	0.49 (0.25, 0.98)	0.043
**Mental Health and Substance Use**					
***Mental health - secondary outcomes, pre-specified***					
Depression score high= more depressed	24.5 (12.4)	18.7 (11.3)	0.003	−4.56 (−7.70, −1.42)	0.004
Trauma experience high= more trauma	18.0 (12.1)	13.8 (10.2)	0.018	−3.12 (−6.68, 0.45)	0.086
Experience of stress due to lack of work high=more stress	12.1 (2.8)	12.6 (2.4)	0.260	0.44 (−0.29, 1.18)	0.239
Experience shame due to lack of work high=more shame	10.6 (2.2)	10.1 (2.7)	0.220	−0.26 (−1.02, 0.50)	0.500
**Mental health - tertiary outcomes, not pre-specified**					
GAD score high=more anxiety	9.5 (5.6)	7.2 (5.0)	0.006	−1.53 (−3.11, 0.05)	0.057
Problem drinking	23 (26.1%)	20 (27.4%)	0.860	0.83 (0.41, 1.68)	0.600
Audit score	0.5 (0.0–4.0)	0.0 (0.0–4.0)	0.490	−0.67 (−1.27, −0.07)	0.030

Livelihoods showed a consistent pattern whereby women in the intervention arm reported improved outcomes (Table 4) as hypothesised. Specifically, women’s earnings in the past 4-weeks were significantly increased, whether treated as a binary (none versus any, aOR2.11, 95%CI 1.04, 4.28) or as a continuous variable (β0.98, 95%CI 0.05, 1.90). Similarly, women’s savings in the past 4-weeks were also significantly higher in the SSCF+ arm (none versus any, aOR3.29, 95%CI 1.14, 9.46; continuous β3.29, 95%CI 1.14, 1.99). Food insecurity in the past 4 weeks was also significantly reduced among the SSCF+ participants (β-0.92, 95%CI -1.64, -0.21). Tertiary livelihood outcomes showed significant improvement, including working in the past three months (aOR2.86, 95%CI 1.34, 6.11), reduced borrowing because of lacking food in the past 4 weeks (aOR0.34, 95%CI 0.15, 0.76) and reduced stealing because of hunger in the past 4 weeks (aOR0.49, 95%CI 0.25, 0.98).

Mental health outcomes among women in the SSCF+ arm improved at endline as was hypothesised, with significant reductions in depressive symptoms (β-4.56, 95%CI -7.70, -1.42) and alcohol consumption (β-0.67, 95%CI -1.27, -0.07). Non-significant reductions were also seen for post-traumatic stress symptoms. Supplementary analyses for showed no major differences to outcomes (S3 and S4 Tables).

#### Men’s outcomes.

At endline among men allocated to SSCF+, past 6-month perpetration of physical IPV was significantly reduced (aOR0.38, 95%CI 0.17, 0.81), with non-significant reductions found in sexual IPV (aOR0.77, 95%CI 0.39, 1.51) and severe IPV (aOR0.86, 95%CI 0.47, 1.56) (Table 5). There were also significant improvements in communication with their partner (β1.94, 95%CI 0.26, 3.61) and non-significant reductions in emotional and economic IPV perpetration (Table 5).

**Table 5:** Men’s outcome measures at endline.

	Control	Intervention	p-value	Model 1	
	**N=76**	**N=82**		**aOR/β (95%CI)**	**p-value**
**Primary Outcomes**					
Physical IPV in past 6m	32 (42.1%)	25 (30.9%)	0.140	0.38 (0.17, 0.81)	0.013
Sexual IPV in past 6m	17 (22.4%)	17 (20.7%)	0.800	0.77 (0.39, 1.51)	0.447
Severe IPV in past 6m	24 (31.6%)	23 (28.0%)	0.630	0.86 (0.47, 1.56)	0.615
***IPV, gender norms - tertiary outcomes, not pre-specified***					
Emotional IPV in past 6m	39 (57.4%)	43 (56.6%)	0.930	0.84 (0.46, 1.56)	0.583
Economic IPV in past 6m	36 (52.9%)	37 (48.7%)	0.610	0.62 (0.27, 1.42)	0.261
Gender inequitable attitudes>= less equitable gender attitudes	26.3 (5.9)	26.6 (5.7)	0.760	1.28 (−0.03, 2.59)	0.055
Controlling behaviour>= more controlling	27.8 (6.4)	28.3 (6.7)	0.650	0.43 (−1.54, 2.40)	0.671
Communication>= better	19.5 (5.6)	21.8 (6.1)	0.019	1.94 (0.26, 3.61)	0.023
**Livelihoods**					
***Livelihoods - Secondary pre-specified***					
Any earnings in past 4 weeks	59 (80.8%)	73 (92.4%)	0.035	3.22 (0.85, 12.18)	0.084
Amount of earnings in past 4 weeks	350 (70–700)	400 (200–1200)	0.100	0.60 (−0.12, 1.33)	0.104
Any savings in past 4 weeks	40 (59.7%)	46 (63.0%)	0.690	1.70 (0.59, 4.86)	0.325
Amount of savings in past 4 weeks	50 (0-400)	50 (0-200)	0.460	0.01 (−0.99, 1.00)	0.991
Food insecurity score high=more insecure	6.9 (2.5)	6.8 (2.3)	0.780	−0.05 (−0.81, 0.70)	0.888
***Livelihoods - Tertiary, not pre-specified***					
Worked in past 3 m	32 (42.1%)	46 (56.8%)	0.066	2.00 (1.06, 3.78)	0.032
Livelihood activities score high=more active	11.4 (4.8)	11.7 (4.8)	0.670	0.14 (−1.43, 1.71)	0.86
How easy to find R200 in emergency	0	0		n/a	
Borrow food or money	25 (33.8%)	16 (19.8%)		0.42 (0.17, 1.06)	0.066
Taken something that was not yours	26 (34.7%)	30 (37.0%)	0.760	1.06 (0.53, 2.13)	0.870
**Mental Health and Substance Use**					
***Mental health - secondary outcomes, pre-specified***					
Depression score high= more depressed	16.6 (8.9)	17.8 (9.1)	0.400	0.27 (−2.29, 2.84)	0.835
Trauma experience high= more trauma	12.1 (8.0)	13.0 (8.1)	0.460	0.86 (−1.08, 2.81)	0.384
Experience of stress due to lack of work high=more stress	11.3 (2.4)	12.0 (2.8)	0.140	0.65 (−0.17, 1.47)	0.123
Experience shame due to lack of work high=more shame	9.7 (2.3)	10.3 (2.3)	0.130	0.42 (−0.33, 1.16)	0.272
**Mental health - tertiary outcomes, not pre-specified**					
GAD score high=more anxiety	6.4 (4.9)	5.7 (4.3)	0.330	−1.07 (−2.19, 0.05)	0.062
Problem drinking	28 (36.8%)	26 (32.1%)	0.530	0.8 (0.38, 1.68)	0.563
Audit score	2.0 (0.0–4.5)	2.0 (0.0–4.0)	0.740	0 (−0.74, 0.74)	0.998

Livelihood outcomes for men in the intervention showed a consistent pattern of improvement. Working in the past three months significantly improved (aOR2.00, 95%CI 1.06, 3.78) and there were non-significant improvements in earnings and savings in the past 4 weeks, and less borrowing because of lack of food. For mental health outcomes, there was no clear pattern of change for men in the intervention arm, compared to the control arm.

Supplementary analyses for men showed similar results, with no changes for adjusting for rural/urban (S3 Table). The per protocol analysis showed similar patterns to the main analysis (S4 Table), but also showed sexual IPV perpetration was significantly less often seen in the intervention arm (aOR0.44, 95%CI 0.19, 1.00) and borrowing because of hunger was significantly reduced (aOR0.27, 95%CI 0.11, 0.69), while stress related to lack of work among men in the intervention arm, increased (β1.06, 95%CI 0.16, 1.97).

## Discussion

This study sought to assess the acceptability and potential for impact on IPV, mental health and livelihoods of the co-developed SSCF+ among young people living in urban informal settlements and rural communities in KwaZulu-Natal, South Africa. Overall, the intervention was generally acceptable, well attended and there was positive qualitative feedback from participants that mostly supported the key new components of the intervention. There was also evidence that the intervention was promising and an impact evaluation trial would be justified, as shown by the changes in many key outcomes in the correct direction supporting the broad model of the intervention. Some further adaptation is recommended after this evaluation.

Among marginalised young people living in challenging settings a key challenge is ensuring that they can attend interventions. In our prior work with young people from informal settlements attendance at 70%+ of sessions was 22% for men and 31% for women, driven by work seeking and, for women, childcare [16]. In this study, attendance at 70%+ of sessions was higher: 53% for men and 81% for women. This improved attendance was likely driven by several factors. First, through delivering the intervention to small friendship groups it was likely friends could support each other’s attendance. Second, the travel stipend of R100/session, was higher than in the original trial, which was R70 (~US$3.80, inflation adjusted). Indeed, given mean past month earnings for women and men was R300 (excluding government grants) this was likely a substantial drawcard. The small-scale nature of this pilot also meant that compared to a large-scale trial, facilitators may have been more able to be flexible in changing session times, and following up with participants who did not attend. Despite these unknowns, the much-improved attendance compared to the original SSCF trial, suggests that SSCF+ is acceptable to young people.

Recruiting and intervening with small friendship groups was well liked and supported the broad intervention approach enabling people to build relationships with friends and share sensitive issues. This also meant participants could talk openly and reflect on their lives, connecting their individual stories with other people’s experiences [22]. However, small friendship groups did cause some challenges around cluster retention, as whole groups would drop out, something unlikely to happen if larger groups were formed. This is likely driven by friends influencing attendance, and ‘exiting as a group’. The study design also meant that some friendship groups were split up as potential participants were too young or too old to be included. Expanding the age range may improve cluster sizes.

The two mixed-gender sessions sought to build communication and empathy between women and men, a key component of effective IPV prevention interventions [20], which while liked, also caused challenges. Studies have often shown that in mixed-gender groups men are more likely to dominate conversations and interrupt women, although this can vary by context and topic of conversation [38]. To counter this, SSCF+ was designed to build women’s confidence and ability to articulate their views, and develop men’s active listening, prior to these mixed-gender groups. However, when meeting each other for the first time, men may have performed a more normative heterosexual masculinity [39], which included disparaging women, to establish themselves hierarchically [40]. While facilitators would have sought to challenge such comments and disrupt these dynamics, facilitators also had to navigate and maintain their identities in these spaces [41]. Strategies to address this in the future would include strengthening facilitator training to support them to push back and establish more equitable dynamics. Moreover, working further with men prior to these mixed sessions on building empathy towards women would be important.

The quantitative findings support a future trial to assess the effectiveness of the intervention at reducing IPV, with consistent patterns of change in the hypothesised direction for primary, secondary and tertiary outcomes. Among men, there was a significant reduction in past 6-month physical IPV perpetration and non-significant reductions for economic IPV and sexual IPV. While similar to the findings in the original SSCF trial, there were indications of larger effect size, in the main analysis the size of reduction of physical IPV (aOR0.34) was larger than in the original trial (aOR0.71), although 95 percent confidence intervals overlap. Additionally, in the per protocol analysis there was a significant reduction in sexual IPV perpetration in this study, which was not seen in the original trial. For women, there was a consistent pattern of reduced IPV in the intervention group, although not significant, and these effect sizes were consistent with other interventions that have shown impact [42], and much larger than the original trial (where they were all around aOR 0.9 [15]).

Women and men who participated in SSCF+ showed improved livelihoods, which were similar to the original SSCF trial [15], and is one of the key mechanisms designed to reduce IPV. The focus of SSCF+ on providing a mixture of livelihood skills, plus critical reflection about livelihoods, and setting small goals around livelihood, all appear important in generating change [43]. The small amount of cash received for travel support, where travel costs were much lower, or negligible, may have contributed to the overall impact of the intervention on livelihoods. While studies have demonstrated positive impacts of cash transfers in reducing IPV perpetration and experience [44], others, particularly short-term transfers in complex settings, have led to less clear outcomes with stress increasing at the point transfers end [45]. Informally, facilitators reported participants used this money for a range of activities, including buying food, paying for children’s school fees and investing in starting businesses. Qualitative research from South Africa on the use of cash transfers found cash used for a variety of purposes, including investing in the future, but also on short-term spends, such as alcohol [46]. Further research on how participants use this cash, and whether this supported increasing livelihood opportunities is required.

There were mixed findings for mental health and substance use. For women the consistent pattern of reductions in measures of poor mental health and alcohol use is promising for future impacts on reductions in IPV. Poor mental health and alcohol use are both recognised risk factors for women’s IPV experience [47,48] and it is assumed that improving these will lead to reductions in IPV. SSCF+ had an explicit focus on improving mental health, including in each session activities designed to support stress reduction, which many young people reported likely. Additionally, when participants told their life stories, there was an emphasis on identifying strengths and how people had overcome challenges, reflecting a collective narrative approach [25]. There may have also been benefit in the intervention focusing on small groups of friends and addressing friendship dynamics, with improvements in friendships also contributing to improvements in mental health.

It is unclear why there was no indication of change in men’s mental health among those in the intervention as qualitatively many reported the sessions and approach as beneficial. Informal discussions with facilitators suggested while men did enjoy the specific activities around stress reduction, some may felt embarrassed practicing these if others could see them, potentially as they did not conform to dominant forms of masculinity [49]. It may also have been that men struggled, particularly in group settings, to discuss emotions, given the strong emphasis among young men in this context of not speaking about emotions [49,50] and thus, developing ‘emotional literacy’ was harder for them and a longer process. Finally, there was a substantial focus in young men’s lives in their complex material circumstances, with previous research emphasising how young men unable to provide and fulfil dominant ideals of masculine success in these spaces, particularly supporting children, family and establishing a home, are described as ‘children’ [39], impacting on their self-esteem and identity as men, and in turn impacting their mental health. While livelihoods improved, it may not have been enough in such a short period to address these intersections. Other research has suggested that men in particular, may benefit from approaches that strengthen their existing positive coping strategies, such as sport [51]. In revising SSCF+ prior to a future trial, it may be beneficial to ensure that there are a range of different strategies provided for young people to reduce stress and improve mental health, which build on their own solutions, such as listening to music, engaging in support and so forth.

There was also no impact on men’s alcohol use. In the original SSCF trial [15], alcohol use was only significantly reduced after 24 months, and it may be that it takes time for change around alcohol to occur than we assessed. We also only used the shorter AUDIT-C scale, which may be less sensitive to smaller changes. It may have been, however, that there remained much resistance in small friendship groups to stopping drinking [52] and a greater focus on challenging alcohol norms is required.

### Limitations

This study has several limitations. The qualitative data were collected post-intervention and often people like to emphasise the positive aspects of interventions, although we note that some areas that did not work so well for all participants were described. The small sample size meant that at baseline there were some significant differences between arms, which could have biased the results. We recognise that sample size was small but it was not our intention to conduct an under-powered RCT and so we sought evidence of promise and consider that this was shown in the results. Participants self-reported outcomes and this could have biased reports, however the lack of change in some indicators suggests this did not happen consistently, if at all. Importantly, the high rates of endline retention meant loss-to-follow-up, and the bias this introduces, was not a major issue. IPV prevention trials would not normally have only 5 months follow-up, and so we do not know what may have happened to outcomes in the longer term.

A broader concern is that SSCF+ as a relatively short intervention – compared to the weight of history and structural inequalities young people live in and navigate through – it cannot and does not address these in anyway. Rather it supports young people to navigate them in slightly less deleterious ways for their health and wellbeing and in turn positively benefiting their partner and friends. However, given these wider concerns, future interventions should consider how through community activism and other mechanisms, structural change can be achieved, which may only benefit individuals in the longer run.

## Conclusion

SSCF+ was acceptable for young people and showed enough promise to justify some light-touch further adaptation and a formal impact evaluation. The intervention requires a few changes to strengthen it, for a trial we would recommend reviewing the inclusion and exclusion criteria, and strengthening facilitation skills to ensure that mixed-gender groups are more effectively led to ensure women are not negatively impacted. Overall, this suggests that a definitive, properly powered evaluation of SSCF+ including a process and economic evaluation is required to assess whether the intervention retains its promise and IPV in this complex setting can be prevented.

## Supporting information

S1 Table Intervention outline. (DOCX)

S2 Table Power calculations. (DOCX)

S3 Table Outcomes adjusting for urban/rural location. (DOCX)

S4 Table Endline outcomes for women and men, using a per protocol analysis (those attending >70% of sessions). (DOCX)

S1 Consort CONSORT checklist. (DOCX)
