# Supplementary material for: Stepping Stones and Creating Futures Plus: A pilot randomised controlled trial of a co-developed intervention with young South Africans
Source: PLOS Glob Public Health. 2025 Apr 23;5(4):e0004494. doi: 10.1371/journal.pgph.0004494 (PMC12017541; doi:10.1371/journal.pgph.0004494)
Supplement: S1 Table — (DOCX) [file pgph.0004494.s001.docx]

| **Supplementary Table 1: Intervention overview**  This provides a short overview of the 15 sessions comprising Stepping Stones and Creating Futures Plus (SSCF+). | |
| --- | --- |
| **Session** | **Topic/Focus** |
| 1 | **Introductions and setting the scene:** the first session helps participants form a peer group (a group of people of the same age, social status and interest). The sessions helps participants explain what they want from the course and to make it clear if we can meet these expectations. It also introduces basic listening, communication and cooperation skills. |
| 2 | **Situating Ourselves:** the second session starts to explore ourselves and our relationships with others, and learn and practice basic communication skills. Start to write life stories. |
| 3 | **Telling My Story**: Participants are provided an opportunity to tell their life story, via ‘The River of Life’ activity. As participants speak about their lives, facilitators help participants identify key moments where participants have shown strength, resilience, skills and other positive qualities. Participants practice attentive listening and conversation skills. |
| 4 | **Telling My Story (continuation from Session 3):** Others are provided with the space to share their stories. |
| 5 | **Understanding my networks (part 1):** The session starts by identifying key social resources participants draw on to survive, with a focus on friendship groups. Challenges of social relationships are also discussed, with a focus on how to problem solve difficulties when these arise. This is backed up by key communication skills, which are practiced in the group. |
| 6 | **Understanding my networks (part 2):** The sixth session supports participants to reflect on how they have used the communication skills learned in the previous session and practice them some more. This is done through reality-based scenarios, which are acted out, and discussed by the group. Other important relationships in people’s lives are identified. |
| 7 | **Experiencing More Positive Intimate Relationships (Part 1)**: The seventh session helps participants to analyse their intimate partnerships and to learn ways of making these more fulfilling and supportive. There is a focus on positive and negative aspects of relationships, sexual intimacy and sex. |
| 8 | **Joint meeting between women’s and men’s groups**: The eighth session brings men and women together to share reflections on gender and relationships, and start to question gender norms. This is focused around discussions of ideal images of men and women. |
| 9 | **Experiencing More Positive Intimate Partnerships (Part 2)**: The ninth session builds on the previous two, but more explicitly and directly tackles questions of power, abuse and violence in intimate relationships. Role plays, discussions and reflection are used to support people to engage. |
| 10 | **Joint meeting between women’s and men’s groups:** The tenth session brings men and women together to discuss reflections on conflict in intimate partnerships. Role plays, sharing different views/perspectives and prepared in the previous session are the central mechanism. |
| 11 | **Making a Living (Part 1)**: The eleventh session introduces the idea of livelihoods and focuses on formal employment. This includes discussing experiences from employment and finding work to discussing and identifying concrete strategies for overcoming barriers to work seeking, and work. |
| 12 | **Making a Living (Part 2):** The twelfth session supports participants to identify income generating opportunities in their communities, using a range of participatory techniques including income generation, mapping opportunities, and planning opportunities. |
| 13 | **Making a Living (Part 3):** The thirteenth session supports participants to analyse how they spend money and to consider the advantages and disadvantages of debt and savings. |
| 14 | **My Possible Self:** The fourteenth session helps participants to set limited, short term and achievable goals across multiple areas of their lives. It includes reflecting back on their life stories (Sessions 3&4) and identifying dreams from there and then identifying concrete, realistic goals which could be achieved in 6-month timeframes. |
| 15 | **Supporting Ourselves**: fifteenth session helps participants to review the course and to proceed with meeting independently if they choose to. |
