# Supplementary material for: Stepping Stones and Creating Futures Plus: A pilot randomised controlled trial of a co-developed intervention with young South Africans
Source: PLOS Glob Public Health. 2025 Apr 23;5(4):e0004494. doi: 10.1371/journal.pgph.0004494 (PMC12017541; doi:10.1371/journal.pgph.0004494)
Supplement: S2 Table — (DOCX) [file pgph.0004494.s002.docx]

**Supplementary Table 2**

**Power calculations for IPV reduction at varying % IPV reduction, and varying ICC, at α=0.05, n (at endline) =160, with 16 clusters per arm and 5 people per cluster**

|  | | | |
| --- | --- | --- | --- |
| IPV-prevalence control | IPV prevalence  intervention | Power (ICC=0.01) | Power (ICC=0.1) |
| 0.30 | 0.15 | 0.61 | 0.57 |
| 0.30 | 0.16 | 0.54 | 0.51 |
| 0.30 | 0.17 | 0.48 | 0.45 |
