# Supplementary material for: Stepping Stones and Creating Futures Plus: A pilot randomised controlled trial of a co-developed intervention with young South Africans
Source: PLOS Glob Public Health. 2025 Apr 23;5(4):e0004494. doi: 10.1371/journal.pgph.0004494 (PMC12017541; doi:10.1371/journal.pgph.0004494)
Supplement: S4 Table — (DOCX) [file pgph.0004494.s004.docx]

Supplementary Table 4: Endline outcomes for women and men, using a per protocol analysis (those attending >70% of sessions)
